# Neurology-Related Research Using the German Disease Analyzer Database: A Narrative Review of Studies Published Between 2020 and 2025

**DOI:** 10.3390/neurosci7020046

**Published:** 2026-04-18

**Authors:** Karel Kostev, Henning Sievert, Marcel Konrad, Christian Tanislav, Jens Bohlken

**Affiliations:** 1 Marburg University, University Hospital, 35043 Marburg, Germany; 2 Epidemiology, IQVIA, 60549 Frankfurt am Main, Germany; 3 Real World Evidence, IQVIA, 60549 Frankfurt am Main, Germany; henning.sievert@iqvia.com; 4 Health & Social, FOM University of Applied Sciences for Economics and Management, 60486 Frankfurt am Main, Germany; 5 Department of Geriatrics, Diakonie Hospital Jung Stilling Siegen, 57074 Siegen, Germany; 6 Faculty of Medicine, Institute of Social Medicine, Occupational Health and Public Health, University of Leipzig, 04109 Leipzig, Germany

**Keywords:** database, Disease Analyzer, routine care research, epidemiology, neurological diseases

## Abstract

Background: The IQVIA Disease Analyzer (DA) database is a major outpatient electronic health record dataset in Germany. Over recent years, it has been increasingly used to study neurological diseases, comorbidities, treatment patterns, and long-term sequelae. We narratively summarized neurology-related studies using the German IQVIA Disease Analyzer (DA) database published since 2020 and to highlight methodological considerations relevant for interpreting DA-based neurological research. Methods: We conducted a narrative review of DA-based studies published between January 2020 and December 2025. PubMed was searched using DA-related keywords and major neurological disease terms. Eligible articles included peer-reviewed cohort, case–control, or descriptive studies using DA outpatient data. Results: The review identified studies covering epilepsy, cerebrovascular outcomes, Parkinson’s disease, dementia, multiple sclerosis, migraine, and sensory disorders. Most used retrospective cohort or nested case–control designs with regression or propensity score methods. Follow-up durations ranged from 3 to 10 years. Results consistently reflected routine care outpatient diagnostic and prescribing patterns. Discussion: Strengths of DA studies include large patient populations, long follow-up, and detailed prescription information. Limitations include reliance on outpatient ICD-10 coding, lack of detailed neurological phenotyping, and potential residual confounding and bias. Conclusions: DA-based analyses generate clinically relevant routine care evidence on neurological conditions in the German outpatient setting. Proper methodological safeguards and complementary data sources are required to contextualize findings for clinical and epidemiological use.

## 1. Introduction

Neurological disorders represent one of the leading causes of morbidity and mortality worldwide. Analyses from the Global Burden of Disease (GBD) studies have shown that neurological conditions collectively account for a substantial and increasing proportion of disability-adjusted life years (DALYs) on a global scale, with a marked rise between 1990 and 2016/2019 [1,2]. Major contributors to this burden include stroke, dementia, epilepsy, Parkinson’s disease (PD), multiple sclerosis (MS), brain and other central nervous system (CNS) cancers, traumatic brain and spinal cord injuries, meningitis, and headache disorders [1,3].

Stroke is one of the largest contributors to global neurological disability. GBD analyses for 1990–2016 and 1990–2019 demonstrated that, despite declines in age-standardized mortality in many regions, the absolute number of incident strokes and stroke survivors continues to increase due to population aging and the growing prevalence of vascular risk factors [4,5]. A separate analysis of lifetime risk reported that in many countries more than one in three adults can expect to experience a stroke during their lifetime, underlining the relevance of long-term prevention and follow-up strategies [6].

Dementia constitutes another major global health challenge. The GBD dementia analyses estimated a marked increase in dementia prevalence between 1990 and 2016, with projections indicating a further tripling of cases by 2050 in the absence of effective preventive measures [7]. Long-term cohort studies such as the Framingham Heart Study have documented changes in dementia incidence over recent decades and highlighted the role of vascular and lifestyle factors in modulating risk [8]. Importantly, dementia is not restricted to older adults. A recent meta-analysis estimated a substantial global prevalence of young-onset dementia, indicating that cognitive impairment in midlife is also a relevant public health issue [9]. More recent GBD-based work further quantified the global burden of Alzheimer’s disease and other dementias from 1990 to 2019 and underscored large regional differences and the contribution of modifiable risk factors [10].

Epilepsy is one of the most common chronic neurological conditions worldwide. The GBD 2016 epilepsy analysis reported marked geographic variation in incidence and prevalence and showed that a substantial part of the burden arises in low- and middle-income countries, although high-income countries also carry a considerable load [11].

Parkinson’s disease is among the fastest-growing neurological disorders in terms of prevalence and associated disability. The GBD 2016 PD analysis demonstrated that the number of people living with Parkinson’s disease more than doubled between 1990 and 2016 [12]. These quantitative findings align with epidemiological observations describing PD as a “pandemic”, driven by aging populations and environmental exposures [13].

Multiple sclerosis also shows increasing prevalence in many regions. The GBD 2016 MS analysis highlighted substantial regional variation in prevalence and incidence and a clear overall increase in MS burden over time [14]. Subsequent age–period–cohort analyses based on GBD 2019 data suggested that these trends are consistent across several world regions and are likely influenced by improved diagnostics, survival, and environmental risk factors [15].

Headache disorders, particularly migraine, are highly prevalent and disabling. A GBD-based analysis of the 2019 data confirmed that migraine remains one of the leading causes of years lived with disability (YLDs) worldwide, especially among young and middle-aged women [16]. The GBD 2016 headache analysis further quantified the burden associated with both migraine and tension-type headache across regions and age groups [3].

Beyond these conditions, several other neurological diseases substantially contribute to global DALYs. GBD 2016 analyses quantified the burden of brain and other CNS cancers, meningitis, and motor neuron diseases, demonstrating considerable mortality and long-term disability associated with each of these disorders [17,18,19]. Traumatic brain and spinal cord injuries were also shown to account for a large share of neurological DALYs, particularly in younger and middle-aged populations [20]. In addition, certain otological conditions such as sudden sensorineural hearing loss (SSNHL) represent important neurological emergencies. Population-based data suggest that SSNHL occurs with an incidence in the range of a few tens of cases per 100,000 persons per year and carries a risk of persistent hearing impairment [21,22].

More recent updates of GBD and related analyses have confirmed that the overall burden of neurological disorders continues to increase between 1990 and 2019, with substantial regional heterogeneity in patterns and trends [23]. Classical neuroepidemiological work has also emphasized that many “common” neurological disorders are more prevalent than previously assumed and often coexist with other chronic diseases [24]. Together, these findings highlight the need for robust real-world evidence (RWE) to complement randomized trials by characterizing diagnostic pathways, treatment patterns, comorbidity profiles, and long-term outcomes in routine care.

In Germany, large outpatient healthcare databases such as the IQVIA Disease Analyzer offer an important opportunity to address these questions. They enable the study of incidence rates, diagnostic and therapeutic strategies, and comorbidities in everyday practice for a broad range of neurological disorders, including stroke and cerebrovascular outcomes, dementia, epilepsy, Parkinson’s disease, multiple sclerosis, headache disorders, and sudden sensorineural hearing loss. In the following sections, we summarize and critically discuss existing Disease Analyzer–based studies on these conditions in order to place their findings in the context of the broader neuroepidemiological literature.

To our knowledge, no recent review has specifically synthesized neurology-related studies based on the German IQVIA Disease Analyzer database while also focusing on the methodological implications of using routine outpatient data in this field. Restricting the review to the period 2020–2025 was intended to capture the most recent phase of DA-based neurological research, characterized by increasing study volume and thematic diversification. Despite the growing number of DA-based studies in neurology, there remains a limited synthesis of their methodological strengths, limitations, and translational implications. This review therefore aims not only to summarize recent studies, but also to critically contextualize their findings within a broader epidemiological framework.

## 2. Review Approach and Evidence Selection

This article follows a narrative review design. Study identification was primarily based on the authors’ comprehensive knowledge of the Disease Analyzer literature accumulated over many years of research with this database. To enhance transparency and ensure completeness, a supplementary PubMed search was performed using combinations of the terms “Disease Analyzer”, “IQVIA”, “Germany”, and specific neurological conditions (e.g., epilepsy, stroke, Parkinson’s disease, dementia, multiple sclerosis, migraine), restricted to the period January 2020 to December 2025.

The PubMed search yielded 380 records when using the term “Disease Analyzer”. Restricting to studies related to neurological diseases resulted in 63 records. After limiting to the period 2020–2025, 39 publications remained, of which 38 were available in English. Of these, 13 studies were excluded because neurological conditions were included only as covariables rather than as primary study outcomes or inclusion criteria. The remaining studies were considered eligible and were supplemented by additional relevant publications known to the authors based on their expertise with the Disease Analyzer database.

Studies were included if they (1) used the German IQVIA Disease Analyzer database, (2) addressed neurological or neuro-related outcomes, (3) were original peer-reviewed research articles, and (4) were published between 2020 and 2025. Studies were excluded if neurological conditions were not a primary focus, if the database used was not the German Disease Analyzer, or if articles were non-original research.

Study selection was guided by relevance to neurology, study design (cohort, case–control, or descriptive analyses), and clarity of outcome definition. Studies were grouped into major disease domains (epilepsy, vascular/traumatic brain disorders, neurodegeneration, demyelinating disease, and sensory/headache disorders).

As this is a narrative review, no formal PRISMA protocol, risk-of-bias scoring, or quantitative synthesis was applied. This approach allows conceptual synthesis but may introduce selection bias, which is acknowledged in the Discussion. Because Disease Analyzer research is conducted across multiple research groups but often focuses on clinically relevant or statistically significant associations, publication bias toward positive findings cannot be excluded. A narrative review differs from a systematic review in that it summarizes and interprets existing studies without applying formal protocol-driven study selection, risk-of-bias assessment, or quantitative synthesis.

## 3. The Disease Analyzer Database as a Platform for Neurology Research

The DA database contains anonymized longitudinal data extracted from German general and specialist practices, including ICD-10 diagnoses, prescriptions, basic demographics, and visit dates. Note: “Disease Analyzer” is the official proprietary name of this outpatient database. The term refers only to the dataset itself and does not imply analytic functions; therefore, the full database name is retained to avoid ambiguity with other IQVIA data products. Its strengths—large sample sizes, long follow-up windows, and routine care data captured from both general practice and specialties such as neurology, ENT, and internal medicine—make it particularly well-suited to addressing questions about disease incidence, comorbidity patterns, and long-term complications.

The DA database currently includes electronic health records from approximately 3–4 million active patients per year. The contributing practices comprise around 3000 German outpatient clinics including general practitioners and specialists (neurology, psychiatry, gynecology, urology, and others). This distribution reflects the structure of the German outpatient sector and ensures broad representativeness across age groups and regions [25].

This article follows a narrative review approach, aiming to summarize and contextualize key DA-based neurological studies published since 2020 without applying systematic review methodology. Most DA studies in neurology use retrospective cohort or case–control designs with propensity score matching or multivariable regression to control for confounders such as age, sex, consultation frequency, and selected comorbidities. Neurologically focused studies published since 2020 can be grouped broadly into those concerning epilepsy, vascular and traumatic brain outcomes, neurodegeneration and cognitive disorders, demyelinating disease, and other neurological manifestations, including headaches and sensorineural hearing loss (Table 1). Where original studies did not report median or IQR follow-up, the tables present the maximum follow-up duration as provided in the source publications.

Overall, the identified studies clustered primarily in epilepsy-related research, neurodegenerative and cognitive disorders, cerebrovascular outcomes, demyelinating disease, and headache or sensory disorders, illustrating both the thematic breadth and the uneven distribution of DA-based neurology research during the review period.

## 4. Epilepsy: Comorbidities, Risk Factors, and Health Care Delivery

Several DA analyses have focused on epilepsy as either an index disease or a long-term complication. A series of studies have examined cardiometabolic risk factors and cardiovascular comorbidities in adults with epilepsy. Using matched cohorts drawn from general practice data, one analysis demonstrated that epilepsy was associated with a higher incidence of subsequently recorded heart failure diagnoses compared with individuals without epilepsy, even after adjusting for traditional cardiovascular risk factors [26]. This finding suggests that epilepsy may serve as a risk marker for subsequent cardiac dysfunction and underscores the need for more aggressive cardiovascular risk management in this population.

In parallel, a series of etiological studies has examined whether common chronic conditions predispose individuals to the subsequent development of epilepsy. A retrospective cohort analysis showed that atrial fibrillation is associated with an increased incidence of epilepsy in adults followed in German practices [27]. Another study with a similar design found that gout was associated with a higher incidence of epilepsy in adjusted analyses, suggesting that systemic inflammation and metabolic disturbance may play a role in epileptogenesis [28]. A third analysis investigated the relationship between body mass index and epilepsy, revealing sex-specific associations between BMI and incident epilepsy that point toward complex interactions between adiposity, vascular risk, and seizure susceptibility [37]. Together, these studies illustrate how the DA database can be utilized to move beyond basic incidence counts and delve into upstream risk constellations.

The link between epilepsy and cerebrovascular disease has also been explored using DA data. A machine learning-based study of patients with late-onset epilepsy found that structured information available in primary care, including vascular comorbidities, consultation frequency, and drug prescriptions, can predict subsequent stroke risk with clinically meaningful accuracy [29]. This work illustrates how structured information available in routine primary care can be used to develop predictive models for subsequent outcomes such as stroke in patients with late-onset epilepsy. Importantly, such models primarily reflect patterns embedded in routinely coded data, including comorbidities, healthcare utilization, and prescribing behavior. As a result, their performance depends on the structure and completeness of these variables and may vary across healthcare settings. Consequently, machine learning-based predictions in this context should be interpreted as reflecting data-driven risk patterns rather than validated clinical prediction tools, and require external validation before broader application.

More recently, the DA database has been used to describe early treatment strategies in newly diagnosed epilepsy, focusing on the frequency and determinants of immediate antiseizure medication initiation in adults [37]. These data show that prescribing decisions are strongly shaped by age, comorbidities, and practice characteristics. They also reveal substantial heterogeneity between practices, pointing to potential disparities in the quality of care and areas in which guideline implementation requires improvement.

## 5. Vascular Brain Events and Their Complications

Cerebrovascular disease and its sequelae have been examined from various angles. One of the earlier neurology-related DA-based studies in the period of interest investigated factors associated with fractures after stroke or transient ischemic attack in an outpatient population [38]. The analysis revealed that patients with a history of stroke or TIA were at a substantially increased risk of subsequent bone fractures. It also highlighted the roles of age, sex, and specific comorbidities such as osteoporosis and dementia in modulating this risk. While the primary outcome was musculoskeletal, the work is also clinically relevant for the field of neurology as it quantifies a major contributor to post-stroke morbidity, suggesting that fracture prevention strategies should be incorporated systematically into secondary prevention following cerebrovascular events.

A more recent study used DA primary care data to examine what happens after an episode of syncope. In a large matched cohort, syncope was associated with a higher six-month incidence of ischemic stroke, brain tumor, epilepsy, anxiety disorders, and cardiac arrhythmia [34]. While the absolute risks remained modest, the pattern of outcomes supports the theory that syncope frequently heralds serious underlying pathology, including neurological disease. From a neurological perspective, these results suggest the need for structured diagnostic pathways after syncope, including appropriate neurological work-up in patients with red-flag features or recurrent events.

## 6. Neurodegeneration and Cognitive Impairment

The DA database has been used extensively to explore associations between systemic conditions and dementia or cognitive decline. One of the key studies relevant to neurology in the last five years investigated whether non-alcoholic fatty liver disease (NAFLD) is an independent risk factor for incident dementia in older adults. In a large matched cohort of patients aged 65 years and older, no significant association was found between NAFLD and all-cause dementia, vascular dementia, or anti-dementia drug prescription after adjusting for shared metabolic risk factors [32]. This negative result is important because it suggests that the increased dementia risk observed in patients with NAFLD in unadjusted analyses can largely be explained by the high prevalence of classic cardiovascular and metabolic comorbidities.

Neurodegeneration has also been examined from the perspective of prodromal manifestations. A neuroepidemiology study used DA data to characterize diagnoses and symptoms in the years before a first Parkinson’s disease (PD) code was documented [31]. Patients later diagnosed with PD had prior documentation of depression, constipation, sleep disturbances and other non-motor symptoms more frequently than matched controls. These findings confirm in routine practice what has long been described in specialist cohorts, namely that PD has a long prodromal phase involving non-motor manifestations. Importantly, the DA study shows that many of these prodromal features are already visible to general practitioners, supporting the idea that risk-stratified screening tools could be implemented at the primary care level.

## 7. Demyelinating Disease and Infectious Risk Factors

One of the most recent DA-based analyses has focused on multiple sclerosis (MS). A large retrospective cohort study investigated whether infectious mononucleosis, as a clinical manifestation of Epstein–Barr virus infection, is associated with an increased incidence of MS in patients followed in German general practices [36]. More than one million individuals contributed person-time to this study, and those with a history of infectious mononucleosis were found to be at significantly higher risk of subsequent MS than their matched controls. The DA-based effect estimates aligned well with those from large prospective cohorts in other countries, lending external validity to the findings.

This work illustrates how DA data, despite the lack of imaging and detailed neurological examination, can replicate key epidemiological observations in neuroimmunology. It also underscores the value of the DA database in addressing questions about relatively rare neurological outcomes, which require very large base populations to achieve adequate statistical power.

## 8. Headache and Sensory Disorders

Headache and migraine have recently been added to the DA neurology portfolio. A 2025 cohort study used DA data from over 30,000 individuals to examine whether a prior diagnosis of heatstroke was associated with a higher incidence of subsequently diagnosed migraine [30]. Heatstroke cases identified in general practice were matched to controls without heatstroke using propensity score matching, and five-year cumulative incidence curves demonstrated that individuals with prior heatstroke were roughly twice as likely to be diagnosed with migraine. This association was consistent in both men and women, as well as in younger and older patients. While residual confounding cannot be ruled out, the study suggests that severe heat-related illness may have long-term neurological sequelae in the form of chronic headache and situates migraine within the broader context of climate-related health effects.

Another application related to neurology concerns sudden sensorineural hearing loss. ENT practice data from the DA database were used to describe routine care pharmacological management patterns of idiopathic sudden sensorineural hearing loss, as well as to explore the associations between different treatment strategies and subsequent outcomes [35]. Although the DA database does not contain detailed audiometric data, the study revealed substantial variability in corticosteroid use and adjunctive therapies, emphasizing the need for more standardized treatment protocols. From a neurological perspective, this kind of analysis is relevant because sudden hearing loss is often considered a neurotological emergency with vascular or inflammatory mechanisms. Table 2 summarizes the main neurological and neuro-related outcomes and effect estimates.

## 9. Strengths of the IQVIA Disease Analyzer Database

The IQVIA Disease Analyzer database has a set of distinct strengths that render it a uniquely valuable resource for epidemiological, pharmacoepidemiological, and health services research in Germany. One core advantage is its representativeness: The database is populated with data from a nationwide panel of general practitioners and specialists, the demographic and regional distribution of which closely mirrors that of the German outpatient sector. This sampling design ensures that findings derived from Disease Analyzer data can be extrapolated meaningfully to the broader population and allows researchers to study routine care patterns without the distortions typically introduced by selective recruitment or survey-based methods.

Another major strength lies in the granularity and clinical relevance of the recorded data. The database contains ICD-10 diagnoses, prescriptions with ATC codes, referral patterns, anthropometric measures, and routine laboratory results. Because the DA database captures diagnoses as they are entered by practicing physicians at the time of care, the data reflect actual clinical decision-making in everyday practice. This level of detail enables nuanced analyses, such as distinguishing between disease incidence, prevalence, progression, and treatment trajectories.

The database also provides longitudinal follow-up, enabling researchers to track patients over many years. This continuity makes the DA database particularly suitable for studying chronic diseases, comorbidity accumulation, long-term outcomes, and time-to-event analyses such as the incidence of complications or treatment failures. The longitudinal structure further supports the use of advanced statistical techniques, including cohort matching, survival analyses, and machine learning approaches applied to temporal data.

Another strength is the accuracy of prescription and diagnosis documentation, which is ensured by direct electronic extraction from physician practice management systems. This reduces the risk of recall bias, transcription errors, and retrospective misclassification. From a pharmacoepidemiological perspective, the database is especially valuable because prescription issuance reflects physicians’ actual prescribing behavior, rather than relying on patient self-reporting or reimbursement-only records.

Additionally, the large sample size of the Disease Analyzer database allows for the study of rare outcomes or small subpopulations. This is crucial in neurological research, where many disorders—such as multiple sclerosis, motor neurone disease, and late-onset epilepsy—have low population prevalence. Access to large numbers of patients within a routine care dataset enables the generation of evidence that would otherwise require costly bespoke cohort studies or national registry linkages.

Finally, because the DA database includes data from neurologists, psychiatrists, and general practitioners, it supports interdisciplinary investigations into the full spectrum of neurological and neuropsychiatric conditions. This facilitates the analysis of pathways of care, referral networks, and comorbidity patterns across disciplines—an area in which Germany lacks comprehensive registries.

## 10. Methodological Considerations and Limitations Specific to Neurological Questions

All findings summarized in this review should be interpreted as associative rather than causal, as they are derived from retrospective observational analyses based on routine care data.

Several common methodological features—and limitations—emerge across these studies. Firstly, all analyses rely on ICD-10 diagnostic codes and prescription data recorded in routine practice. For neurological diseases, this implies that precise phenotyping (e.g., seizure type, epilepsy syndrome, disability scores in MS, or cognitive test results in dementia) is unavailable. Misclassification may occur when symptoms such as dizziness or headache are coded non-specifically [32]. In addition, the implications of coding-based case identification extend beyond general misclassification. Diagnostic latency is a relevant concern, particularly for slowly progressive conditions such as Parkinson’s disease and dementia, where symptoms may precede formal coding by several years. Consequently, incident diagnoses in DA studies may reflect a mixture of true incident and delayed prevalent cases. Furthermore, the absence of clinical severity measures (e.g., Hoehn–Yahr stage, cognitive test scores) limits the ability to stratify patients according to disease stage, which may lead to heterogeneity in outcome definitions and effect estimates. These limitations are not uniform across neurological disorders and must be interpreted in a disease-specific context.

Secondly, the DA database lacks direct information on imaging, electroencephalography, cerebrospinal fluid analyses, and detailed neuropsychological test batteries, all of which are central to modern neurology. Studies therefore have to use proxies, such as specialist diagnoses, the prescription of disease-specific drugs, or patterns of repeat visits, to infer disease severity or confirm outcomes such as epilepsy or MS [26,36]. Diagnostic validity is an important limitation in DA-based neurology research. ICD-10 coding accuracy varies between disorders: epilepsy and multiple sclerosis diagnoses made by neurologists are generally reliable, whereas prodromal Parkinson’s disease, mild cognitive disorder, dizziness, or headache codes may be inconsistently used in primary care. Inter-physician variability—including differences between general practitioners and neurologists—can contribute to misclassification. The absence of imaging, EEG, CSF, and neuropsychological data amplifies this uncertainty and affects some conditions (e.g., dementia subtypes, prodromal PD) more than others (e.g., MS coded by specialists).

Thirdly, several epidemiological biases may influence DA-based neurological studies. Surveillance and diagnostic-intensity bias may inflate associations in conditions where affected individuals have more frequent consultations (e.g., gout or AF preceding epilepsy; chronic disease preceding cognitive codes). Reverse causation is possible in studies where prodromal symptoms precede neurological disease (e.g., constipation or depression before Parkinson’s disease). Treatment-comparison studies may be affected by immortal-time bias and time-lag bias, especially when exposure definitions rely on prescription records. Finally, in older cohorts, the competing risk of death may attenuate or distort dementia incidence estimates. These biases should be considered when interpreting the effect sizes summarized in this review. Although many studies applied propensity score matching or regression, balance diagnostics, standardized mean differences, and model performance metrics were not consistently reported in the original publications and are therefore not uniformly available in this review. Effect sizes summarized in tables represent point estimates; confidence intervals vary by study and should be considered when interpreting clinical relevance. Predictive modeling approaches, such as the stroke prediction model in late-onset epilepsy, serve prognostic rather than causal purposes and lack external validation or calibration data, limiting their immediate clinical applicability.

Fourthly, disease-specific considerations also limit interpretability. Epilepsy comprises heterogeneous syndromes with differing etiologies, yet DA data do not distinguish seizure types or epilepsy subtypes. Prodromal Parkinson’s disease analyses may be affected by circular reasoning because non-motor symptoms used for case identification also form part of prodromal PD criteria. Mild cognitive disorder codes in primary care may reflect health-seeking behavior rather than objective decline. For multiple sclerosis, outpatient ICD-10 codes may underrepresent diagnostic confirmation typically established in neurology clinics or hospitals, requiring cautious interpretation when comparing with registry-based studies.

Fifthly, although propensity score matching and multivariable regression are widely used to control for confounding, residual confounding by lifestyle factors (smoking, alcohol, physical inactivity), genetic predisposition, and socioeconomic variables remains a concern. This is particularly relevant in analyses linking systemic conditions such as gout, NAFLD or heatstroke to neurological outcomes such as epilepsy, dementia or migraine [28,30,32].

Sixthly, for several conditions, the DA database only captures patients seen in outpatient settings. While this is very suitable for chronic disease management, it may miss acute neurological events that are managed exclusively in hospital and never coded in primary care, such as certain types of strokes, acute demyelinating episodes, or severe encephalitis. Researchers must therefore interpret incidence estimates with caution and, where possible, complement DA analyses with data from hospital or registry sources. Table 3 shows the limitations of DA-based studies and how they can be mitigated or interpreted.

Finally, a substantial proportion of DA-based neurology studies originate from teams with overlapping authorship, including individuals affiliated with IQVIA, the database provider. While data extraction processes are standardized and automated, this overlap may influence topic selection or interpretation. Independent replications using DA data or comparisons with external datasets remain limited and represent an important future need to ensure interpretive neutrality.

Selected studies and their key methodological characteristics and limitations are summarized in Table 4.

## 11. Cross-Cutting Methodological Insights from DA-Based Neurology Studies

Across neurological disease domains, Disease Analyzer-based studies show a number of recurring analytical patterns that extend beyond individual conditions. Rather than being disease-specific, many findings are shaped by structural characteristics of routine outpatient data. In particular, associations often reflect differences in healthcare utilization, diagnostic practices, and treatment pathways, which influence how and when conditions are recorded in the database.

A consistent strength across studies is the ability to detect relative differences and temporal patterns in large, unselected patient populations under real-world conditions. This allows robust estimation of associations within routine care and supports the identification of clinically relevant comorbidity constellations.

At the same time, these studies share common inferential boundaries. They are well-suited for descriptive epidemiology and hypothesis generation but remain limited in their ability to capture disease complexity, establish causal relationships, or provide mechanistic insights. Interpreting results across disease areas, therefore, requires an understanding that many observed patterns arise from the structure of the data rather than from disease-specific biological mechanisms alone.

Framing DA-based evidence within these overarching principles helps to integrate findings across neurological domains and to position routine care data appropriately within the broader hierarchy of epidemiological evidence.

## 12. Future Directions

This review is intended to support clinical pharmacologists, neurologists, and therapeutics researchers in understanding how outpatient routine care data can inform neurological epidemiology and treatment-related questions. The German IQVIA Disease Analyzer database is particularly relevant for evaluating medication use, long-term safety signals, and comorbidity patterns in a routine care setting.

Generalizability beyond Germany is limited by differences in healthcare system structure, coding practices, and access pathways. DA data reflect the German outpatient sector, which differs from claims-based systems in the US, GP-gatekeeper systems such as those in the UK, and nationwide neurology registries in Scandinavian countries. Comparisons across systems must therefore account for structural differences in coding incentives, consultation frequency, and referral patterns.

Taken together, the DA-based studies published in the last five years demonstrate that routine primary care and specialist care data can make a substantial contribution to neurological epidemiology. Regarding epilepsy, future studies could combine DA data with hospital discharge data to capture acute seizures and status epilepticus, and link risk factor profiles with hard outcomes such as mortality or disability. Existing studies on cardiovascular comorbidities and stroke risk in epilepsy indicate that combined neurological and cardiological risk models could be developed and validated using the DA database [2,5].

Regarding neurodegenerative and cognitive disorders, the negative association between NAFLD and dementia, adjusted for metabolic risk factors [32], indicates that the DA database can be used to both refute and support hypothesized risk relations. A natural next step would be to examine the effects of treatments such as statins, antihypertensives, and antidiabetic agents on dementia risk in high-risk groups, leveraging the large sample sizes and long follow-up times available in DA once again.

In demyelinating disease, the strong association between infectious mononucleosis and incident MS [36] could be further investigated by exploring the interplay between EBV infection, vitamin D supplementation, smoking habits and other autoimmune conditions. Similarly, the heatstroke–migraine analysis opens up a broader agenda concerning climatically triggered neurological disease, which could be linked to temporal patterns of high-temperature episodes and regional variations in coding at the practice level [6].

Methodologically, there is significant potential to apply advanced causal inference and machine learning approaches to the DA database data. The stroke-risk prediction model in late-onset epilepsy [29] is an example of how such techniques can be employed. Similar models could be developed to predict the conversion of prodromal PD to manifest disease, mild cognitive disorder to dementia, or infectious mononucleosis to MS. However, the limitations of observational routine data must always be taken into account.

A unifying methodological implication across these studies is that DA data enable exploration of neurological outcomes using large-scale, routine care outpatient information, while highlighting the constraints of routine coding. Together, these findings illustrate how administrative–clinical data can contribute to hypothesis generation, risk stratification research, and descriptive epidemiology, but they also underscore the need for cautious causal interpretation and integration with more deeply phenotyped cohorts.

## 13. Conclusions

This narrative review summarizes neurology-related studies published since 2020 using the German IQVIA Disease Analyzer database. The DA database provides routine care, outpatient diagnostic, and prescription information suitable for describing disease incidence, comorbidity profiles, medication use, and longitudinal outcomes. Because the data originate from routine outpatient coding, they lack detailed neurological phenotyping and therefore support descriptive epidemiology rather than causal inference.

From a clinical and translational perspective, DA-based evidence reflects real-world patterns of diagnosis, treatment, and comorbidity in unselected patient populations. Such data can help identify high-risk groups, characterize treatment pathways, and generate hypotheses relevant to clinical research. However, these findings should be interpreted as complementary to evidence from randomized controlled trials and prospective cohort studies. While DA-based analyses provide valuable insights into routine care, they are not designed to establish causality or to directly inform clinical guidelines without corroboration from other study designs.

When interpreted with appropriate attention to confounding, coding validity, and bias, the Disease Analyzer database represents a valuable resource for clinical epidemiology and pharmacological research in neurology.

## Figures and Tables

**Table 1:** Overview of key Disease Analyzer neurology studies.

First Author (Year)	Neurological/Neuro-Related Outcome	Exposure/Main Question	Design	N (Patients)	Setting	Reported Maximum Follow-Up (Years) *
Doege et al., 2021 [26]	Heart failure in late-onset epilepsy	Epilepsy vs. no epilepsy	Retrospective cohort	19,292	GP	10
Doege et al., 2022 [27]	Atrial fibrillation in late-onset epilepsy	Atrial fibrillation vs. no atrial fibrillation	Retrospective cohort	149,362	GP	10
Doege et al., 2023 [28]	Epilepsy in gout	Gout vs. no gout	Retrospective cohort	224,964	GP	10
Kostev et al., 2021 [29]	Ischemic stroke in late-onset epilepsy	Clinical predictors & AEDs	Retrospective cohort + ML	11,466	GP	8
Kostev et al., 2025 [30]	Migraine after heatstroke	Heatstroke vs. no heatstroke	Retrospective cohort	34,764	GP	5
Bohlken et al., 2022 [31]	Prodromal presentations of Parkinson’s disease	Primary-care symptom patterns	Nested case–control	20,000	GP	10
Labenz et al., 2021 [32]	Dementia in nonalcoholic fatty liver disease (NAFLD)	NAFLD vs. no NAFLD	Retrospective cohort	44,634	GP	10
Yaqubi et al., 2024 [33]	Multiple sclerosis in IBD	IBD (CD/UC) vs. no IBD	Retrospective cohort	29,868	GP	10
Gümbel et al., 2023 [34]	Stroke, epilepsy, and other disorders in syncope	Syncope vs. no syncope	Retrospective cohort	128,032	GP	Six months
Seidel et al., 2025 [35]	Pharmacotherapy of idiopathic sudden sensorineural hearing loss	Treatment patterns over time	Cross-sectional	96,824	ENT specialist	10
Loosen et al., 2022 [36]	Multiple sclerosis in Infectious mononucleosis	Infectious mononucleosis vs. no infectious mononucleosis	Retrospective cohort	32,116	GP	10
Pfeifer et al., 2022 [37]	Body mass index and the incidence of epilepsy	Different BMI categories	Retrospective cohort	822,071	GP	10
Tanislav & Kostev, 2020 [38]	Factors associated with fractures after stroke	Fractures vs. no fractures	Case–control	36,795	GP	5

All studies in Table 1 were based on the German IQVIA Disease Analyzer database. Abbreviations: GP = general practitioner; ENT = ear, nose, and throat; BMI = body mass index; T2D = type 2 diabetes; IBD = inflammatory bowel disease; CD = Crohn’s disease; UC = ulcerative colitis; AED = antiseizure drug. * Note: Median/IQR follow-up was not available in most original DA publications.

**Table 2:** Main neurological and neuro-related outcomes and effect estimates (examples).

Exposure–Outcome Pair	Outcome Category	Typical Effect Size	Key Clinical Message
Epilepsy → heart failure	Cardiovascular/neurological comorbidity	HR 1.5–1.6	Epilepsy associated with higher HF incidence, particularly in younger patients
Epilepsy → ischemic stroke	Cerebrovascular complication	HR ~1.4	Vascular comorbidities drive most of the excess stroke risk
Prodromal PD symptoms	Neurodegeneration	OR 1.5–3.0	Constipation, depression, sleep and pain disorders precede PD diagnosis
Heatstroke → migraine	Headache/pain	HR ~2.3	History of heatstroke markedly increases 5-year migraine risk
IBD → MS	Neuro-immunological comorbidity	HR ~2.1–2.4	CD and UC both associated with higher subsequent MS risk

Abbreviations: HR = hazard ratio; OR = odds ratio; PD = Parkinson’s diseaseHF = heart failure; MS = multiple sclerosis.

**Table 3:** Limitations and mitigation and interpretation strategies.

Limitation	Mitigation and Interpretation Strategy
Lack of imaging and detailed neurological examination data	Linkage to disease-specific registries and careful case definition validation
Potential diagnostic miscoding or under-recording in primary care	Use of repeated codes, specialist confirmations, and sensitivity analyses with stricter algorithms
No direct information on lifestyle (smoking, alcohol, physical activity)	Use of proxy diagnoses (COPD, obesity, alcohol-related liver disease) and careful confounder discussion
Residual confounding in non-randomized treatment comparisons	Active comparator new user designs and high-dimensional PS methods where feasible
Limited capture of hospital-only events (e.g., acute severe stroke)	Focusing on long-term outcomes and using referral or prescription patterns as indirect markers

Abbreviations: COPD = chronic obstructive pulmonary disease.

**Table 4:** Methodological characteristics and key limitations of selected Disease Analyzer neurology studies.

Study	Key Methodological Issue	Potential Bias	Interpretation Note
Doege et al., 2021 [26]	No clinical severity data	Residual confounding	Association may reflect comorbidity burden
Kostev et al., 2021 [29]	ML model without external validation	Overfitting	Predictive performance may not generalize
Bohlken et al., 2022 [31]	Prodromal symptoms used for case identification	Reverse causation	Early symptoms may be part of disease process
Labenz et al., 2021 [32]	Limited lifestyle variables	Unmeasured confounding	Metabolic factors may explain associations
Yaqubi et al., 2024 [33]	No imaging confirmation	Misclassification	MS diagnosis may vary across practices
Gümbel et al., 2023 [34]	Short follow-up	Detection bias	Early events may be overrepresented

## Data Availability

This is a review article without original data to share.
